# Long Noncoding RNA HOTAIR Modulates MiR-206-mediated Bcl-w Signaling to Facilitate Cell Proliferation in Breast Cancer

**DOI:** 10.1038/s41598-017-17492-x

**Published:** 2017-12-08

**Authors:** Wei Ding, Jin Ren, Hui Ren, Dan Wang

**Affiliations:** 1 Department of General Surgery, The Second Hospital of Jilin University, Changchun, 130041 China; 2 Department of Respiratory medicine, The Second Hospital of Jilin University, Changchun, 130041 China; 3 Department of Colorectal Surgery, The Second Hospital of Jilin University, Changchun, 130041 China; 4 Department of Breast Surgery, The Second Hospital of Jilin University, Changchun, 130041 China

## Abstract

LncRNA HOX transcript antisense RNA (HOTAIR) is involved in lots of cancers. The pro-survival protein Bcl-w is frequently found in cancer development. However, the effect of HOTAIR on Bcl-w in breast cancer is not well documented. In this study, we first evaluated the correlation between HOTAIR level and Bcl-w expression in clinical breast cancer tissues. We observed that the expression levels of Bcl-w were much higher in the breast cancer samples than that in their paired noncancerous tissues. Moreover, the levels of HOTAIR were positively associated with those of Bcl-w in clinical breast cancer samples. As expected, we observed that HOTAIR was able to up-regulate the expression of Bcl-w in breast cancer cells. Mechanistically, we found that miR-206 was capable of inhibiting the expression of Bcl-w by directly binding to the 3′UTR of Bcl-w mRNA. Interestingly, HOTAIR could increase the expression of Bcl-w through sequestering miR-206 at post-transcriptional level. Functionally, our data showed that HOTAIR-induced Bcl-w by miR-206 facilitated the proliferation of breast cancer cells. Thus, we conclude that HOTAIR up-regulates Bcl-w to enhance cell proliferation through sequestering miR-206 in breast cancer. Our finding provides new insights into the mechanism of breast cancer mediated by HOTAIR.

## Introduction

Only up to 2% of human genome is translated into proteins^1–4^. Non-coding RNAs (ncRNAs) consist of small ncRNAs and long ncRNAs (lncRNAs). LncRNAs are composed of more than 200 nucleotides. Studies shows that lncRNAs participate in some cellular processes, including chromatin modification, genomic reprogramming, RNA processing, cell proliferation, cell cycle and apoptosis^5–12^. As a subset of lncRNAs, large intergenic non-coding RNAs (lincRNA) are transcribed from genomic locuses among protein-coding loci^13,14^. LincRNA HOX transcript antisense RNA (HOTAIR) can mark the homeobox D gene cluster (HOXD) for transcriptional repression. Highly expressed HOTAIR has been implicated in many types of malignancies^15–21^. HOTAIR is able to interact and recruit polycomb repressive complex 2 (PRC2) to induce HOXD gene silencing^22^. HOTAIR competes with BRCA1 for binding to EZH2 to regulate various gene expressions^23^. HOTAIR represses the expression of Wnt inhibitory factor 1 (WIF-1) or phosphatase and tensin homolog (PTEN) to participate in EMT of cancers^24,25^. HOTAIR also regulates matrix metalloproteinases to play roles in tumor invasion^26,27^. However, the underlying mechanism of HOTAIR in breast cancer development need to be further investigated.

Bcl-w (also known as Bcl-2-like protein 2) belongs to bcl-2 family^28^. Bcl-w promotes cell migration and invasion by modulating multiple factors in cancers^29–31^. Bcl-xL is able to induce cell resistance in breast cancer therapy^32^. Bcl-w and Akt1 targeted by miR-133b modulates proliferation and apoptosis of bladder cancer cells^33^. Bcl-w and survivin targeted by miR-203 is involved in chemosensitivity of bladder cancer^34^. MiR-335 inhibits proliferation and invasion via Bcl-w in clear cell renal cell carcinoma (ccRCC)^35^. Bcl-w ablation is capable of enhancing mitotic cell death^36^. Blocking of Bcl-w and Bcl-xL is able to induce apoptosis of senescent cells^37^. Myc-induced miR-15 family members regulating Bcl-w involves in B-cell Lymphoma and overexpressed Bcl-w can serve as a biomarker for diagnosis in lymphomagenesis^38,39^. The function of Bcl-w in HOTAIR-mediated breast cancer is still unexplored.

In this study, we seek to explore whether lincRNA HOTAIR enhances the development of breast cancer through miR-206 targeting Bcl-w. Interestingly, we find that HOTAIR increases Bcl-w expression via sequestering miR-206 at post-transcription level, leading to the promotion of breast cancer growth. Our finding takes a further step into the mechanism of lincRNA HOTAIR-mediated breast cancer growth.

## Results

### HOTAIR is positively associated with Bcl-w in human breast cancer samples

**Figure 1:**
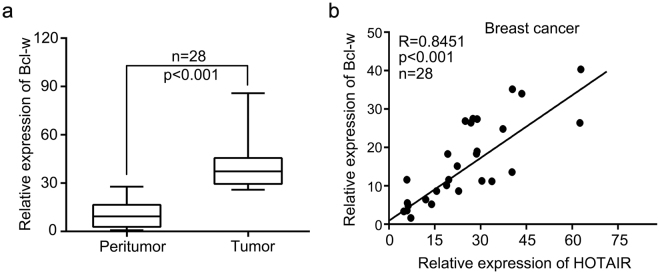
HOTAIR is positively associated with Bcl-w in human breast cancer samples. (**a**) Bcl-w expression was detected by qRT-PCR assay in human breast cancer tissues and their paired noncancerous tissues (Wilcoxon’s signed-rank test). (**b**) Correlation between HOTAIR and Bcl-w was analyzed by qRT-PCR assay in human breast cancer samples (Pearson’s correlation coefficient, R = 0.8451, p < 0.001).

Accumulating evidence demonstrates that HOTAIR plays important roles in cancers^15–21^. Given that Bcl-w is an important pro-survival protein, we speculated that Bcl-w might be involved in HOTAIR-mediated breast cancer progression. To explore the significance of Bcl-w in breast cancer, we detected Bcl-w levels through qRT-PCR assay in human breast cancer tissues and their paired noncancerous tissues. We found that Bcl-w was overexpressed in 28 clinical breast cancer tissues (Fig. 1a, Wilcoxon’s signed-rank test, p < 0.001). Furthermore, qRT-PCR analysis demonstrated that HOTAIR expression was positively correlated with Bcl-w expression in clinical breast cancer tissues (Pearson’s correlation, R = 0.8451, p < 0.001, Fig. 1b). Our data indicate that HOTAIR overexpression is associated with highly expressed Bcl-w in breast cancer.

### In breast cancer cells HOTAIR induces Bcl-w expression

**Figure 2:**
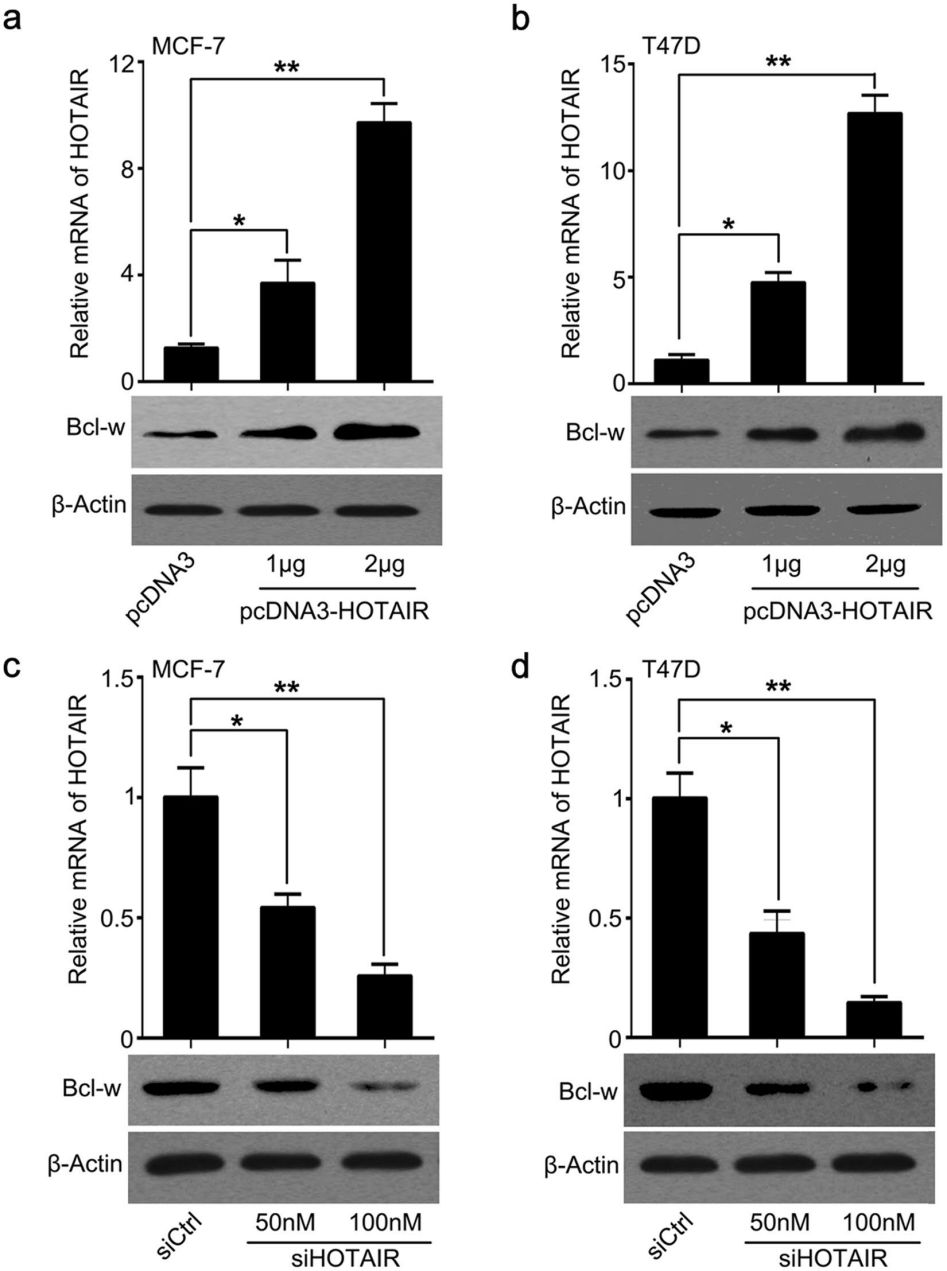
In breast cancer cells HOTAIR induces Bcl-w expression. (**a**,**b**) The modulation of HOTAIR on Bcl-w was determined by qRT-PCR assay and Western blot analysis in breast cancer MCF-7 and T47D cells. (**c**,**d**) The effect of HOTAIR siRNA on Bcl-w expression was investigated by Western blot or qRT-PCR assay. Statistical significant differences are indicated: **p < 0.01, *p < 0.05, Student’s *t*-test.

Taken a step further, we investigated the effect of HOTAIR on Bcl-w in breast cancer cells. The data showed that HOTAIR remarkably increased Bcl-w expression in MCF-7 and T47D cells. The overexpression efficiency of HOTAIR was verified by qRT-PCR analysis in cells (Fig. 2a,b). In contrast, HOTAIR RNA interference resulted in Bcl-w reduction in MCF-7 and T47D cells. Meanwhile, the interference efficiency of siHOTAIR was validated (Fig. 2c,d). Collectively, our data support that HOTAIR is capable of inducing Bcl-w in breast cancer cells.

### MiR-206 suppresses Bcl-w expression *via* directly targeting Bcl-w mRNA 3′UTR

**Figure 3:**
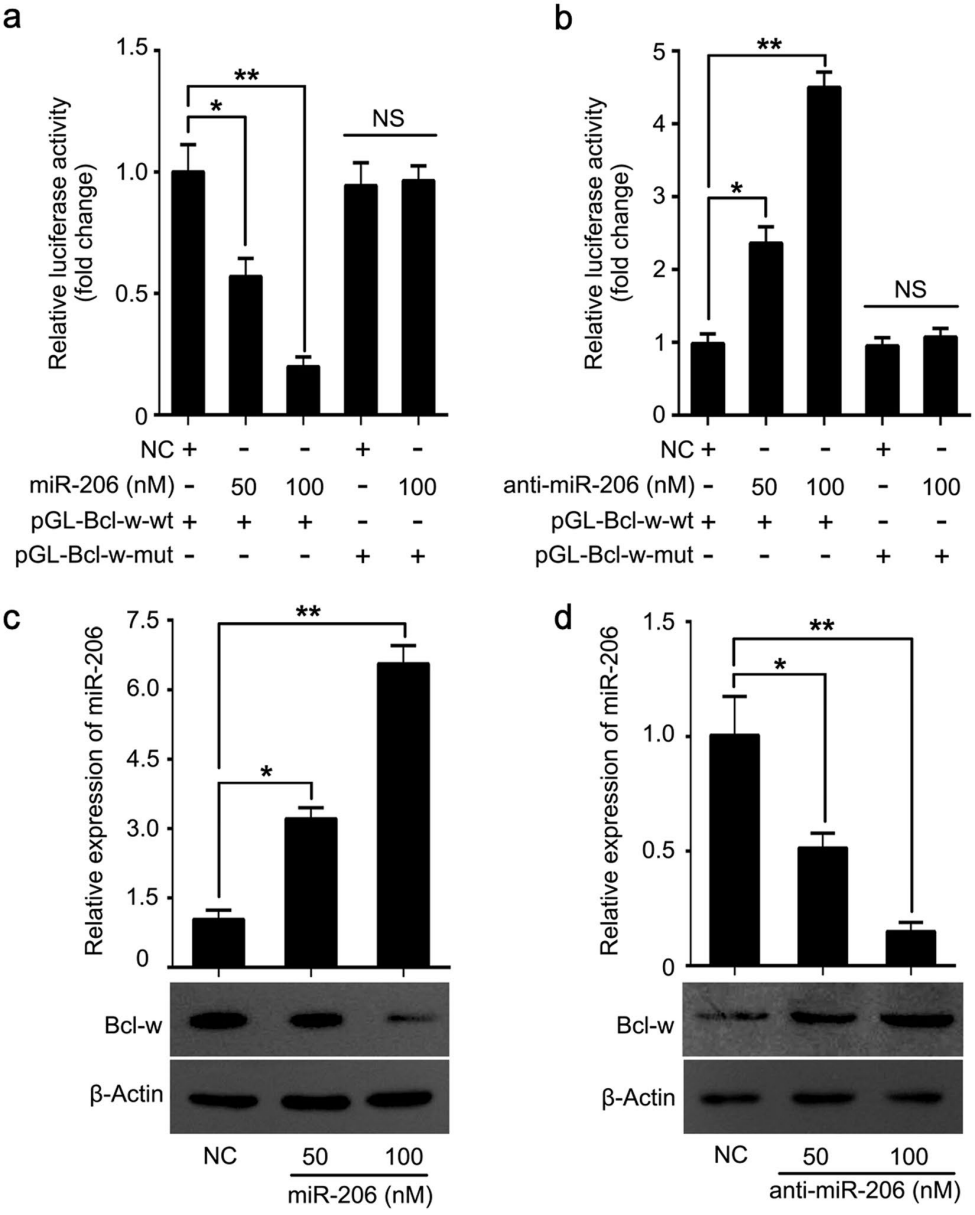
MiR-206 suppresses Bcl-w expression *via* directly targeting Bcl-w mRNA 3′UTR. (**a**,**b**) In MCF-7 and T47D cells regulation of miR-206 (or anti-miR-206) on pGL-Bcl-w-wt and pGL-Bcl-w-mut was tested by using luciferase reporter gene assay. (**c**,**d**) Effect of miR-206 (or anti-miR-206) on Bcl-w expression was examined by Western blot. The level of miR-206 was analyzed by qRT-PCR assay after the cells were transfected with miR-206 or anti-miR-206. Statistical significant differences are indicated: **p < 0.01, *p < 0.05, No Significance (NS), Student’s *t*-test.

Given that lncRNAs can act as a molecular sponge to sequester miRNAs, we speculated that HOTAIR might modulate Bcl-w through miRNAs. We first predicted the miRNAs which might interact with HOTAIR through starBase v2.0 (http://starbase.sysu.edu.cn/). One of candidates, miR-206, peaked our interests most for its important roles in breast cancer. Furthermore, we identified that miR-206 could potentially target 3′UTR of Bcl-w mRNA using TargetScan (http://www.targetscan.org/ Fig. 3a). Based on the bioinformatics prediction, we cloned wild-type and mutant miR-206 binding site in Bcl-w mRNA 3′UTR into pGL3-control, termed pGL-Bcl-w-wt and pGL-Bcl-w-mut. Luciferase reporter gene assay demonstrated that miR-206 could dose-dependently inhibit the luciferase activities of pGL-Bcl-w-wt in MCF-7 cells but not pGL-Bcl-w-mut (Fig. 3a). Conversely, anti-miR-206 increased the luciferase activities of pGL-Bcl-w-wt in a dose-dependent manner, but pGL-Bcl-w-mut failed to work (Fig. 3b), implying that miR-206 can directly target Bcl-w mRNA 3′UTR. Then, we revealed that miR-206 was capable of dose-dependently depressing Bcl-w expression (Fig. 3c), while in anti-miR-206-transfected cells the opposite data was obtained (Fig. 3d). Our results show that miR-206 inhibits Bcl-w expression via directly targeting Bcl-w mRNA 3′UTR.

### HOTAIR is capable of elevating Bcl-w expression through inhibiting miR-206

**Figure 4:**
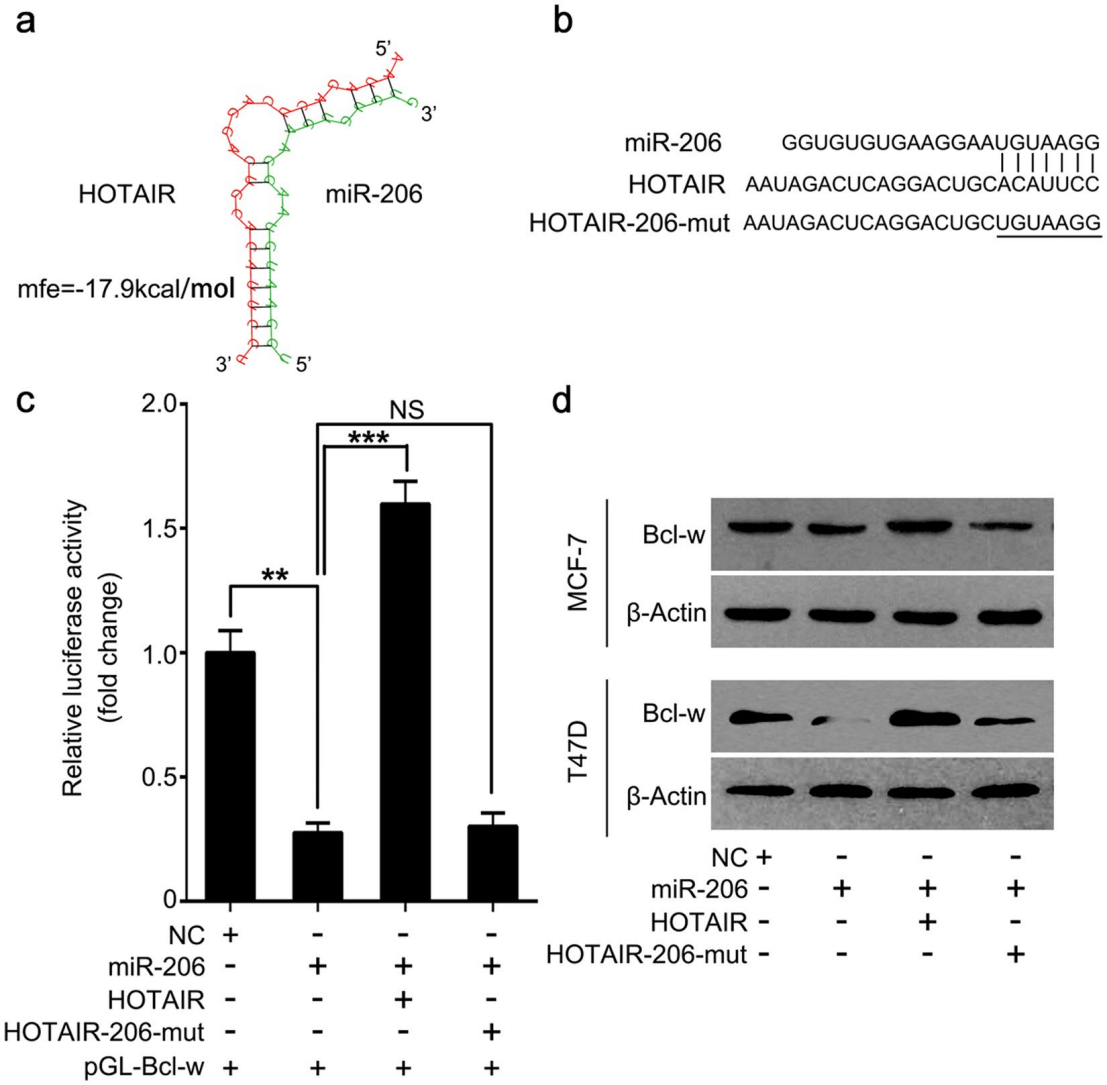
HOTAIR is capable of elevating Bcl-w expression through inhibiting miR-206. (**a**,**b**) Bioinformatics prediction of interaction of HOTAIR with miR-206 through complementary base-pairs was shown. The mutant of sequences of HOTAIR binding to miR-206 (2275–2299) was indicated. (**c**) Luciferase activities of pGL-Bcl-w were analyzed by luciferase reporter gene assay in miR-206 and/or HOTAIR (or HOTAIR-206-mut) transfected MCF-7 cells. (**d**) In miR-206 and/or HOTAIR (or HOTAIR-206-mut) treated cells, Bcl-w expression was assessed by Western blot. ***p < 0.001, **p < 0.01, No Significance (NS), Student’s *t*-test.

Next, we clarified the sponge modulation of HOTAIR on miR-206. We predicted the interaction between HOTAIR and miR-206 by using Bielefeld Bioinformatics Service (http://bibiserv.techfak.uni-bielefeld.de/rnahybrid/submission.html) (Fig. 4a). Bioinformatics analysis revealed that HOTAIR could potentially interact with miR-206 through complementary base-pairing reactions (Fig. 4b). In addition, we constructed the mutant of HOTAIR (HOTAIR-206-mut) with a seven nucleotides substitution in miR-206 binding site. Luciferase reporter gene assay manifested that HOTAIR abrogated the inhibition of pGL-Bcl-wt luciferase activities induced by miR-206 in MCF-7 cells, whereas HOTAIR-206-mut had no effect (Fig. 4c). In addition, we found the similar effect of HOTAIR on Bcl-w expression by Western blotting in MCF-7 and T47D cells (Fig. 4d). Our data imply that HOTAIR can increase Bcl-w expression by sequestering miR-206.

### MiR-206/Bcl-w signal mediates HOTAIR-accelerating cell proliferation in breast cancer

**Figure 5:**
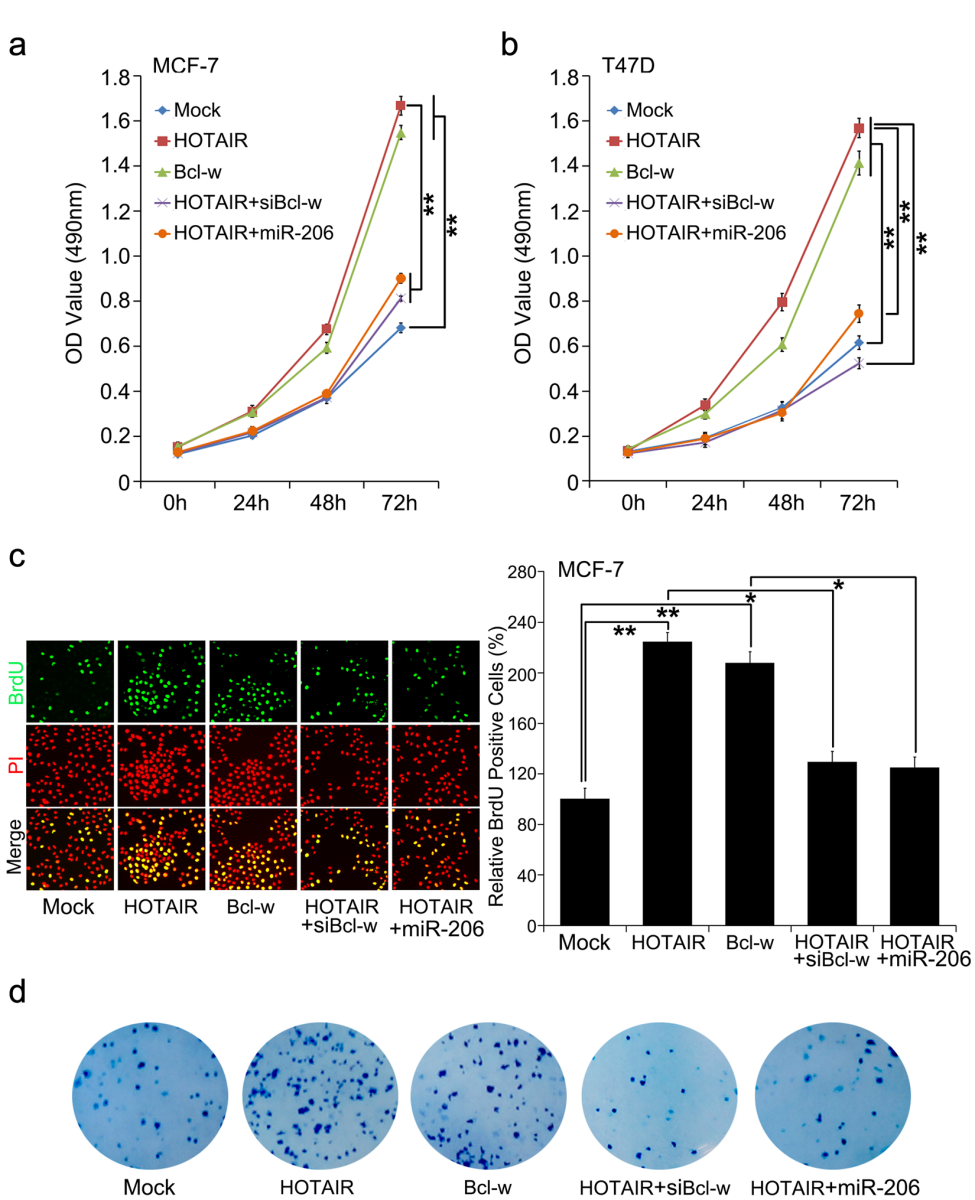
MiR-206/Bcl-w signal mediates HOTAIR-accelerating cell proliferation in breast cancer. (**a**–**c**) Proliferation was tested by MTT and BrdU incorporation assays in MCF-7 or T47D cells treated with the indicated plasmids or siRNAs. (**d**) Colony formation of MCF-7 cells was counted post-transfection with the indicated plasmids or siRNAs.

To uncover whether miR-206/Bcl-w signal participates in HOTAIR-mediated breast cancer cell proliferation, we performed MTT and BrdU incorporation assays in MCF-7 or T47D cells. Our finding revealed that HOTAIR or Bcl-w enhanced MCF-7 and T47D cell proliferation, while ecoptic miR-206 expression or silencing of Bcl-w could inhibit the cell proliferation (Fig. 5a–c). Furthermore, our data showed that HOTAIR or Bcl-w led to increase in colony formation of MCF-7 cells. However, miR-206 introduction or siBcl-w could abolish the augmentation of colony formation induced by HOTAIR (Fig. 5d). Taken together, we conclude that HOTAIR promotes cell proliferation through regulating miR-206/Bcl-w in breast cancer.

## Discussion

LncRNAs play crucial roles in chromatin modification, genomic reprogramming, RNA processing, cell proliferation, cell cycle and apoptosis^5–12^. LincRNAs are a subset of lncRNAs. As the first lincRNA identified to regulate genes at a distance, HOTAIR is overexpressed in lots of cancers, such as breast, gastric, lung, esophageal and liver cancer^40^. Bcl-2 family member Bcl-w promotes cell proliferation, migration, invasion and drug-resistance in cancers^29–34^. The regulation of HOTAIR on Bcl-w in breast cancer development is unexplored. Here, we are wondered whether HOTAIR is part of Bcl-w regulation process in breast cancer.

To uncover the modulation of HOTAIR on Bcl-w in the development of breast cancer, we evaluated the correlation between HOTAIR and Bcl-w in human breast cancer samples. We observed highly expressed Bcl-w in clinical breast cancer tissues. Furthermore, a positive correlation of HOTAIR and Bcl-w was determined in breast cancer samples. Interestingly, we revealed that in breast cancer cells HOTAIR markedly induced Bcl-w expression. Then, we tried to explore the underlying mechanism of HOTAIR elevating Bcl-w. Increasing evidence has highlighted the significance and molecular mechanisms of miR-206, as a tumor suppressor, in many cancers. MiR-1 and miR-206 can target c-Met to depress rhabdomyosarcoma development^41^. MiR-206 targets notch3 to induce the inhibition of Hela cell migration^42^. MiR-206 targeting KLF4 is involved in colon cancer^43^. MiR-206 leads to inhibition of cell proliferation and invasion through regulating VEGF in laryngeal cancer^44^. According to the reports, miR-206 was down-regulated ERα positive breast cancer and ectopic expression of miR-206 could decrease ERα expression through targeting ERα mRNA 3′UTR in human MCF-7 breast cancer cells^45,46^. Accordingly, we used ER positive breast cancer MCF-7 and T47D cells to investigate the regulation of miR-206 on Bcl-w and found that in these cell lines the introduction of miR-206 led to the inhibition of Bcl-w expression via directly targeting the 3′UTR of Bcl-w mRNA.

Given that lncRNAs can act as miRNAs sponge to promote the development of cancers, we presumed that HOTAIR might contribute to Bcl-w elevation by sequestering miRNAs. A recent report reveals that HOTAIR in breast cancer cell lines including ER positive breast cancer MCF-7 cells is higher than those in immortalized breast HBL-100 cells^47^. Another study shows that HOTAIR can regulate P53/Akt/JNK signaling to affect proliferation, migration and invasion of MCF-7 cells^48^. We predicted that miR-206 could potentially interact with HOTAIR. Next, we examined whether HOTAIR could block the decrease of Bcl-w mediated by miR-206 in ER positive breast cancer MCF-7 and T47D cells. We observed that HOTAIR rescued miR-206-inhibted Bcl-w. It suggests that HOTAIR can elevate the expression of Bcl-w through the sponge of miR-206 at post-transcription level. Although our findings were mainly from ER positive breast cancer cells, the relationship between the classification and the main factors (HOTAIR, Bcl-w and miR-206) still need more evidence to prove. Functionally, HOTAIR accelerated cell proliferation through miR-206 targeting Bcl-w in breast cancer. A previous survival analysis demonstrated that high HOTAIR expression in primary breast tumors was significantly associated with worse prognosis independent of prognostic markers and this association was even stronger when looking only at ER positive breast tumor samples^49^. However, another report manifested that HOTAIR as a poor prognostic indicator in ER-negative patients was restricted to node-positive patients and could be a marker for lymphatic metastases rather than hematogenous metastases in ER-negative patients^50^. Our data indicate the accelerating proliferation function of elevated HOTAIR/Bcl-w by inhibiting miR-206 in ER positive breast cancer. It would be a valuable work for us to make in-depth and meticulous investigation of this association between HOTAIR and survival or prognosis in breast cancer in the future. Therapeutically, HOTAIR and Bcl-w may serve as targets for breast cancer.

Overall, here we illustrate a novel mechanism by which HOTAIR modulates cell proliferation via miR-206 targeting Bcl-w in breast cancer. Our finding indicates that HOTAIR is capable of inducing Bcl-w in breast cancer. Mechanistically, miR-206 can decrease the Bcl-w expression through targeting Bcl-w mRNA 3′UTR. Strikingly, HOTAIR can sponge miR-206, leading to increase of Bcl-w expression. Thus, we conclude that HOTAIR up-regulates Bcl-w through sequestering miR-206 at post-transcriptional level to enhance the breast cancer progression. We gain novel insights into the understanding of breast cancer progression induced by HOTAIR.

## Materials and Methods

### Patient samples

Freshly frozen breast cancer tissues and paired noncancerous tissues used in this study were obtained from the Second Hospital of Jilin University (Changchun, China). Patient information was summarized in Supplementary Table S1. All patients were diagnosed with breast cancer and gave written consents. Research ethics committee at the Second Hospital of Jilin University (Changchun, China) approved study protocol. All experiments were performed strictly in accordance with relevant guidelines and regulations. For experiments involving human participants including the use of tissue samples, informed consent had been obtained.

### Cell lines

Breast cancer MCF-7 and T47D cells were maintained in RPMI Medium 1640 (Invitrogen, USA) with 100 U/ml penicillin/streptomycin and 10% FBS.

### Quantitative real-time PCR (qRT-PCR)

Total RNA from tissues or cultured cells was used to do reverse transcription by SuperScript™ IV Reverse Transcriptase (ThermoFisher Scientific, USA). To test miR-206 expression, poly (A) polymerase (Ambion, USA) was applied to polyadenylate total RNA. The qRT-PCR assay was applied using TransStart Top Green qPCR SuperMix (TransGen Biotech, China). The PCR reaction was evaluated using melting curve analysis. Relative transcription alteration was evaluated as 2^−ΔΔCt^ ^51^. GAPDH was used to normalize HOTAIR and Bcl-w. The level of miR-206 was normalized to U6 expression. Primers were shown in Supplementary Table S2.

### Western blot

Lysed cell samples were loading to run SDS-PAGE. Polyvinylidene fluoride (PVDF) membranes with protein samples were incubated with primary antibodies and then second antibodies. The following primary antibodies were used: anti-Bcl-w (Cell Signaling Technology, USA) and anti-β-actin (NeoMarkers, USA). The immune complexes were detected by ECL Western Blotting Substrate (Solarbio, Beijing, China).

### Luciferase reporter gene assay

Cells were plated at 3 × 10^4^/well on 24-well plates. Twenty-four hours later, the cells were co-transfected with the pRL-TK plasmid (Promega, USA) and indicated plasmids or miRNAs. At 48 hour post-transfection, cells were lysed to test luciferase activities. pRL-TK was used to normalize the results.

### Cell proliferation assay

MCF-7 cells were plated at 1000 cells/well onto 96-well plates. After transfection cell proliferation was evaluated by MTT assays once per day for three days.

### BrdU incorporation assay

MCF-7 cells were seeded on 6-well plate and were grown overnight before transfection. Fresh medium containing 10 μmol/L BrdU (Sigma, USA) was used to incubate all groups for 4 hours prior to immunofluorescence staining with mouse anti-BrdU antibody. After fixation with 4% paraformaldehyde in PBS, the cells were incubated overnight with a mouse anti-BrdU antibody (NeoMarkers, USA) and then treated with fluorescein isothiocyanate (FITC)-conjugated goat anti-mouse IgG (Dako, Glostrup, Denmark). Propidium iodine (PI) (Sigma) (50 μg/mL) was used to stain nuclei as the control to all cells in each group. The labeling index was expressed as the number of positively labeled nuclei/total number of nuclei.

### Colony formation assay

For colony formation analysis, treated cells at 1000 cells/well were seeded in 6-well plates with complete medium for 14 days. After methanol fixation and methylene blue staining, colonies were imaged and counted.

### Statistical analysis

All experiments were performed in triplicate. Student’s *t*-test for independent groups was used to compare mean values (±standard deviation, SD) to assess statistical significance: ***p < 0.001, **p < 0.01 and *p < 0.05. Pearson’s correlation coefficient was applied to analyze correlation between HOTAIR level and Bcl-w expression in breast cancer samples.

### Ethical approval

All patients gave written consents. Research ethics committee at the Second Hospital of Jilin University (Changchun, China) approved study protocol. All experiments were performed strictly in accordance with relevant guidelines and regulations. For experiments involving human participants including the use of tissue samples, informed consent had been obtained.

## Electronic supplementary material

Supplementary Dataset 1
