# Supplementary material for: Long Noncoding RNA HOTAIR Modulates MiR-206-mediated Bcl-w Signaling to Facilitate Cell Proliferation in Breast Cancer
Source: Sci Rep. 2017 Dec 8;7:17261. doi: 10.1038/s41598-017-17492-x (PMC5722884; doi:10.1038/s41598-017-17492-x)
Supplement: Supplementary file 1 — Supplementary Dataset 1 [file 41598_2017_17492_MOESM1_ESM.doc]

**Long noncoding RNA HOTAIR modulates miR-206-mediated Bcl-w signaling to facilitate cell proliferation in breast cancer**

Wei Ding^1^, Jin Ren^2^, Hui Ren^3^, Dan Wang^4^

^1^Department of General Surgery, The Second Hospital of Jilin University, Changchun 130041, China; ^2^Department of Respiratory medicine, The Second Hospital of Jilin University, Changchun 130041, China; ^3^Department of Colorectal Surgery, The Second Hospital of Jilin University, Changchun 130041, China; ^4^Department of Breast Surgery, The Second Hospital of Jilin University, Changchun 130041, China

Correspondence and requests for materials should be addressed to D.W. (email: [wangdan79@126.com](mailto:wangdan79@126.com))

**Supplementary Table S1**. Clinical characteristics of breast tissues.

| **No.** | **Age** | **Sex** | **Organ** | **Pathology diagnosis** | **Grade** |
| --- | --- | --- | --- | --- | --- |
| 01 | 46 | F | Breast | Little nonspecific infiltrating ductal carcinoma | I |
| 02 | 63 | F | Breast | A little nonspecific infiltrating ductal carcinoma | I |
| 03 | 54 | F | Breast | Nonspecific infiltrating ductal carcinoma | I |
| 04 | 44 | F | Breast | Nonspecific infiltrating ductal carcinoma | II |
| 05 | 41 | F | Breast | Nonspecific infiltrating ductal carcinoma | I |
| 06 | 65 | F | Breast | Nonspecific infiltrating ductal carcinoma | I |
| 07 | 53 | F | Breast | Nonspecific infiltrating ductal carcinoma | I-II |
| 08 | 44 | F | Breast | Little nonspecific infiltrating ductal carcinoma | I |
| 09 | 29 | F | Breast | Nonspecific infiltrating ductal carcinoma | I-II |
| 10 | 55 | F | Breast | Nonspecific infiltrating ductal carcinoma | I |
| 11 | 63 | F | Breast | Nonspecific infiltrating ductal carcinoma | I |
| 12 | 44 | F | Breast | Nonspecific infiltrating ductal carcinoma | II |
| 13 | 39 | F | Breast | Nonspecific infiltrating ductal carcinoma | II |
| 14 | 68 | F | Breast | Little nonspecific infiltrating ductal carcinoma | I-II |
| 15 | 50 | F | Breast | Little nonspecific infiltrating ductal carcinoma | II |
| 16 | 47 | F | Breast | Nonspecific infiltrating ductal carcinoma | I |
| 17 | 48 | F | Breast | Nonspecific infiltrating ductal carcinoma | II |
| 18 | 38 | F | Breast | Nonspecific infiltrating ductal carcinoma | II |
| 19 | 48 | F | Breast | Nonspecific infiltrating ductal carcinoma | II |
| 20 | 33 | F | Breast | Nonspecific infiltrating ductal carcinoma | II-III |
| 21 | 34 | F | Breast | Nonspecific infiltrating ductal carcinoma | II |
| 22 | 46 | F | Breast | Little nonspecific infiltrating ductal carcinoma | II |
| 23 | 43 | F | Breast | Nonspecific infiltrating ductal carcinoma | II |
| 24 | 61 | F | Breast | Nonspecific infiltrating ductal carcinoma | I-II |
| 25 | 41 | F | Breast | Nonspecific infiltrating ductal carcinoma | I |
| 26 | 58 | F | Breast | Nonspecific infiltrating ductal carcinoma | II |
| 27 | 57 | F | Breast | Nonspecific infiltrating ductal carcinoma | I |
| 28 | 46 | F | Breast | Nonspecific infiltrating ductal carcinoma | II |

Note: “-” in Grade means no grading available.

**Supplementary Table S2.** **List of primers used in this paper.**

| Primer | Forward Primer (5′-3′) | Reverse Primer (5′-3′) |
| --- | --- | --- |
| **Primers for cloning**  Bcl-w CCCCATGGCGACCCCAGCCTC CTGAAT TCTCACTTGCTAGCAAAAAAG  HOTAIR-206-wt  Forward: AAATAGACTCAGGACTGCTGTAAGGTTGTGTAGGTTGTGTGTG  Reverse: CACACACAACCTACACAACCTTACAGCAGTCCTGAGTCTATTT  HOTAIR-206-mut  Forward: AAATAGACTCAGGACTGCACATTCCTTGTGTAGGTTGTGTGTG  Reverse: CACACACAACCTACACAACCTTACAGCAGTCCTGAGTCTATTT | | |
| **Primers for Bcl-w 3′UTR**  wt GCTCTAGATTCCTCAAGGAGAAAACATTCCC GGGGGCCGGCC CAAGGCCAGCTGCAGAACTG  mut GCTCTAGATTCCTCAAGGAGAAAGTTAAGGG GGGGGCCGGCC CAAGGCCAGCTGCAGAACTG  **Primers for qRT-PCR** | | |
| HOTAIR  Bcl-w | CAAACAGAGTCCGTTCAGTGTC  GGCGCACCTTCTCTGATCTG | GTGGATTCCTGGGTGGGT  GTGGTTCCATCTCCTTGTTGACA |
| GAPDH  miR-206 | ATCACCATCTTCCAGGAGCGA  CCACACACTTCCTTACATTCCA | CCTTCTCCATGGTGGTGAAGAC  GCGAGCACAGAATTAATACGAC |
| U6 | CTCGCTTCGGCAGCACA | AACGCTTCACGAATTTGCGT |
| **siRNA sequences** | | |
| siHOTAIR | GAACGGGAGUACAGAGAGAUU |  |
| siBcl-w | GCAGACUUUGUAGGUUAUATT |  |
| siCtrl | UUCUCCGAACGUGUCACGU |  |
